# *Milnesium minutum* and *Milnesium sandrae*, two new species of Milnesiidae (Tardigrada, Eutardigrada, Apochela)

**DOI:** 10.3897/zookeys.580.6603

**Published:** 2016-04-12

**Authors:** Giovanni Pilato, Oscar Lisi

**Affiliations:** 1 Department of Biological, Geological and Environmental Sciences, University of Catania, Via Androne 81, 95124, Catania, Italy

**Keywords:** Tardigrada, Milnesiidae, new species, Sicily, Hawaiian Archipelago

## Abstract

Two new species of *Milnesium* are described, *Milnesium minutum* **sp. n.** from Sicily and *Milnesium sandrae* **sp. n.** from the Hawaiian Archipelago. The body size of *Milnesium minutum* is the smallest of the known species of the genus. The stylet supports are inserted on the buccal tube at 63–66% of its length and the claws have a [3-3]-[3-3] configuration. *Milnesium sandrae* has stylet supports inserted on the buccal tube at 58–60.5% of its length, a [3-3]-[3-3] claw configuration, and the percent ratio between the secondary claw and primary claw length on legs I–III (78.6%–85.5%) clearly higher than on legs IV (70.5%–71.4%). With the description of these two new species, the number of species in the genus is increased to 31.

## Introduction

For 150 years, the genus *Milnesium* was considered monospecific. Realizing that the individual variability of some characters of Eutardigrada was not as wide as believed for a long time, Binda and Pilato (1990) described a second species of the genus: *Milnesium brachyungue* Binda & Pilato, 1990. Subsequently, various authors described many more species.

In this paper, two new species are described: one, *Milnesium minutum* sp. n., from two Sicilian localities and the other, *Milnesium sandrae* sp. n., from Hawai’i Island (Hawaiian Archipelago).

## Material and methods

All studied specimens were mounted in polyvinyl lactophenol. Measurements, in micrometers (µm), and photomicrographs were made under x100 oil immersion, using a Leica Phase Contrast Microscope equipped with “Canon S40” digital camera and Adobe Photoshop Elements 2.0 digital imaging software. The *pt* index (Pilato 1981) is the percent ratio between the length of a structure and the length of the buccal tube. In Milnesiidae, the length of the buccal tube is measured from the anterior margin of the stylet sheaths to the caudal end, including the flexible portion (Tumanov 2006). We measured only specimens that were aligned to provide accurate morphometric measurements; for this reason, when only a small population is available, only few specimens are suitable for measurement. Though this prevents the assessment of statistical analyses, provided the morphological characters are clearly indicative of speciation, this method avoids the sometime questionably large ranges within statistical analyses caused by imprecise measurements. Claw length refers to the maximum length of the external, primary claws correctly oriented with neither bent nor abnormally straight apices. Configuration of the number of claw points on secondary claws (claw configuration) is given according to Michalczyk et al. (2012b).

In addition to the literature descriptions of many species, the following species (deposited in the Binda & Pilato collection) have been examined for comparison: *Milnesium brachyungue* Binda & Pilato, 1990; *Milnesium eurystomum* Maucci, 1991; *Milnesium antarcticum* Tumanov, 2006; *Milnesium asiaticum* Tumanov, 2006; *Milnesium longiungue* Tumanov, 2006.

## Results

### Milnesium minutum sp. n.

Taxon classificationAnimaliaApochelaMilnesiidae

http://zoobank.org/F90A2415-9C36-4D42-BFF1-C21AE5CE5D20

Fig. 1 , Table 1

#### Type locality.

Sicily, Moio Alcantara, Contrada Rinazzo 37°54'04"N, 15°03'08"E.

#### Material examined.

Moio Alcantara: Contrada Rinazzo (holotype and one paratype: (slide No. 4127) from a moss sample on rock collected by Dr. R. Catanzaro (Catania) (April 1986); Noto: Contrada Volpiglia, (one paratype, slide No. 3238) from a moss sample collected on a dry wall by Mr. S. Di Stefano (Catania) (February 1980).

#### Type repository.

Holotype and two paratypes are deposited in the Binda and Pilato Collection (slides Nos. 4127 and 3238), Museum of the Department of Biological, Geological and Environmental Sciences, University of Catania, Sicily.

#### Specific diagnosis.

Body of small size (up about 300 µm in the specimens found); colourless; cuticle smooth; eye spots present; six peribuccal and two lateral papillae present; mouth terminal with six triangular peribuccal lamellae with basal stripes; stylet supports inserted on the buccal tube at about 63–66% of its length; claws of the *Milnesium* type with a [3-3]-[3-3] configuration; primary claws with thin accessory points; secondary claw bases each with a rounded basal thickening (lunule); a long cuticular bar present under claws I–III.

#### Description of the holotype.

**Figure 1.**
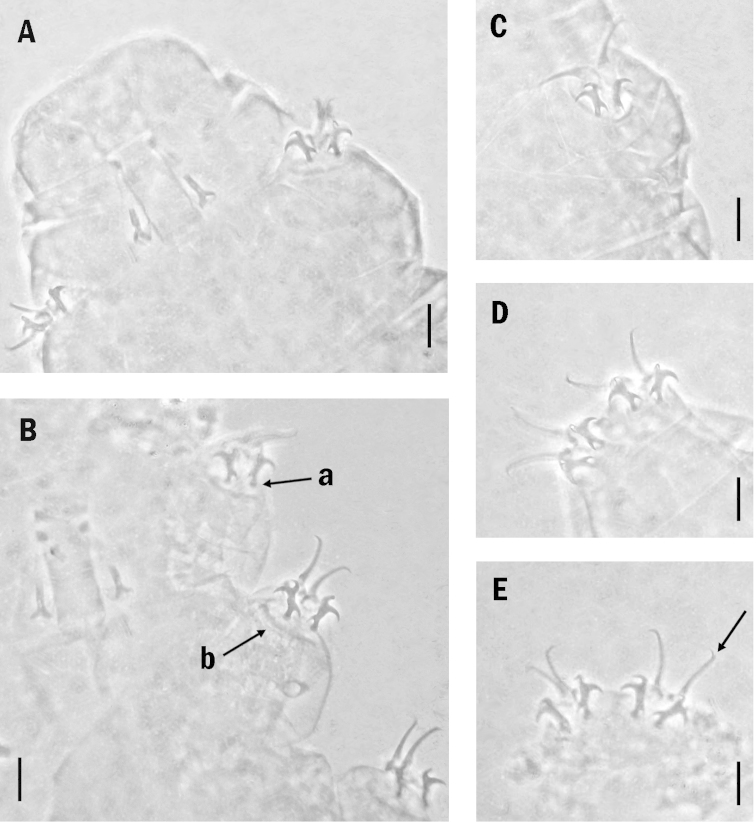
**A–D** *Milnesium minutum* sp. n. (holotype). **A** bucco-pharyngeal apparatus **B** Claws of the second pair of legs; arrow ‘a’ indicates a claw basal thickening (lunule); arrow ‘b’ indicates the long cuticular thickening **C** Claws of the third pair of legs **D** claws of the hind legs **E** Claws of the hind legs of a paratype (slide No. 3238) where the accessory points are visible (arrow). Scale bars: 10 µm.

Body colourless, 288 µm long; cuticle smooth without pseudopores, reticulum, tubercles or gibbosities; eye spots present. Six peribuccal and two lateral papillae present. Bucco-pharyngeal apparatus of the *Milnesium* type (Fig. 1A) (rigid buccal tube without ventral lamina, apophyses for the insertion of the stylet muscles in the shape of very short and flat ridges symmetrical with respect to the frontal plane and without caudal processes; pharyngeal bulb elongated, pear-shaped and without apophyses, placoids or septulum); six triangular peribuccal lamellae present with basal stripes. Wide stylet furcae triangular in shape (Fig. 1A).

Buccal tube cylindrical, 25.7 µm long; the external width at the level of the stylet supports insertion point is 10.9 µm (*pt* = 42.4). Stylet supports short, inserted on the buccal tube at 65.9% of its length.

Claws of the *Milnesium* type (Fig. 1), secondary claw branches with three points: configuration [3-3]-[3-3]. Primary claws on legs II, 11.3 µm long (*pt* = 44.0) and secondary claw, 8.0 µm (*pt* = 31.1); primary claws on legs III, 11.8 µm long (*pt* = 45.9); secondary claw, 8.5 µm long (*pt* = 33.1); primary claws on legs IV, 13.1 µm long (*pt* = 51.0), secondary claw, 8.6 µm (*pt* = 33.5). The secondary claw length is 70.8% of the primary claw length on legs II, 72.0% on legs III and 65.6% on legs IV.

Primary claws with thin accessory points (Fig. 1E arrow); each secondary claw base with rounded basal thickening (lunule) (Fig. 1B, arrow a); a long cuticular bar is present under the claws I–III (Fig. 1B arrow b).

Eggs not found.

#### Remarks.

The paratypes are similar to the holotype in both qualitative and quantitative characters (Table 1).

**Table 1.** Measurements in µm, *pt* index values relative to some structures, and percent ratio between secondary claw and primary claw lengths of the holotype and two paratypes of *Milnesium minutum* sp. n. Also the differences between maximum and minimum values of some characters are given.

Slide nomber Measurements	*Milnesium minutum* sp. n.						
	4127 Moio Alcantara Contrada Rinazzo paratype		4127 Moio Alcantara Contrada Rinazzo holotype		3238 Noto Contrada Volpiglia paratype		Difference between Max.-Min. values
	µm	*pt*	µm	*pt*	µm	*pt*	
Body length	284	-	288	-	?	-	
Buccal tube length	25.8	-	25.7	-	26.4	-	
Buccal tube width	10.9	*42.2*	10.9	*42.4*	10.2	*38.6*	
Stylet supports insertion point		*65.5*		*65.9*		*63.0*	2.9
Primary claw I	10.1	*39.1*	?	?	?	?	
Secondary claw I	7.3	*28.3*	?	?	?	?	
Secondary: primary claw I ratio	72.3%		?		?		?
Primary claw II	10.9	*42.2*	11.3	*44.0*	11.7	*44.3*	
Secondary claw II	7.6	*29.5*	8.0	*31.1*	8.3	*31.4*	
Secondary: primary claw II ratio	69.7%		70.8%		70.9%		1.2
Primary claw III	11.7	*45.3*	11.8	*45.9*	11.7	*44.3*	
Secondary claw III	8.2	*31.8*	8.5	*33.1*	8.5	*32.2*	
Secondary: primary claw III ratio	70.1%		72.0%		72.6%		2.5
Primary claw IV	13.1	*50.8*	13.1	*51.0*	13.2	*50.0*	
Secondary claw IV	8.7	*33.7*	8.6	*33.5*	9.1	*34.5*	
Secondary: primary claw IV ratio	66.4%		65.6%		68.9%		3.3

#### Etymology.

The specific name *minutum* (*minutus* = small) refers to the small body size.

#### Differential diagnosis.

Eight species of *Milnesium* with six peribuccal lamellae and a [3-3]-[3-3] claw configuration are known with a smooth cuticle: *Milnesium brachyungue* Binda & Pilato, 1990; *Milnesium eurystomum* Maucci, 1991; *Milnesium asiaticum* Tumanov, 2006; *Milnesium antarcticum* Tumanov, 2006; *Milnesium longiungue* Tumanov, 2006; *Milnesium zsalakoae* Meyer & Hinton, 2010; *Milnesium barbadosense* Meyer & Hinton, 2012 and *Milnesium bohleberi* Bartels, Nelson, Kaczmarek & Michalczyk, 2014.

**Figure 2.**
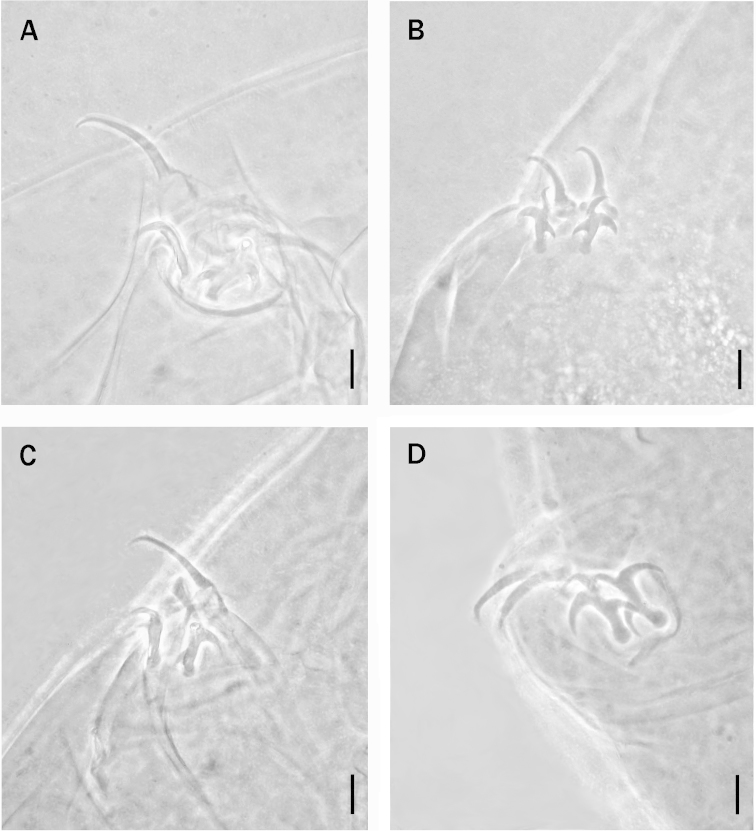
**A** Claws of the third pair of legs of *Milnesium asiaticum*. **B** Claws of the first pair of legs of *Milnesium brachyungue* **C** Claws of the first pair of legs of *Milnesium longiungue* **D** Claws of the second pair of legs of *Milnesium antarcticum*, Scale bars: 10 µm.

*Milnesium minutum* sp. n. differs from all these species in having a smaller body size, and other character detail indicated in the following comparisons. We noticed that the three specimens we attributed to *Milnesium minutum* sp. n. are in particular very similar to *Milnesium asiaticum* and, considering the body size, it was necessary to determine whether they were three young specimens of *Milnesium asiaticum* or belonged to a different species. Three facts have to be stressed: a) we collected the specimens attributed to the new species in two different localities. b) We examined and measured specimens of the 15 species of *Milnesium* present in the collection of Binda & Pilato, and we noticed that for each species in all cases the buccal tube width *pt* index values for smaller specimens were lower than larger specimens. Specimens of the new Sicilian species with 300 µm body length have buccal tube width *pt* values that are similar to (or slightly higher than) those of *Milnesium asiaticum*, which have a body length more than twice as long (Tables 1 and 2). c) *Milnesium minutum* sp. n. differs from *Milnesium asiaticum* in having wider buccal tube with respect to the body length; a lower posterior primary claw *pt* ratio, and a slightly higher percent ratio between the secondary claw and primary claw lengths on legs III and IV (Tables 1–2; Figs 1C, D and 2A). These facts led us to conclude that the three *Milnesium minutum* sp. n. specimens were not young examples of *Milnesium asiaticum* but, independent of body size, belonged to a distinct species.

**Table 2.** Measurements in µm, *pt* index values relative to some structures, and percent ratio between secondary claw and primary claw lengths of a paratype of *Milnesium asiaticum*, the holotype of *Milnesium brachyungue*, and a paratype of *Milnesium longiungue*.

Slide nomber Measurements	*Milnesium asiaticum*		*Milnesium brachyungue*		*Milnesium longiungue*	
	5105 paratype		3940 holotype		5103 paratype	
	µm	*pt*	µm	*pt*	µm	*pt*
Body length	685	-	801	-	747	-
Buccal tube length	54.0	-	59.8	-	46.6	-
Buccal tube width	22.1	*40.9*	23.7	*39.6*	22.1	*47.4*
Stylet supports insertion point		*63.8*		*69.8*		*62.3*
Primary claw I	21.8	*40.4*	13.9	*23.2*	22.5	*48.3*
Secondary claw I	15.1	*28.0*	12.4	*20.7*	14.2	*30.5*
Secondary: primary claw I ratio	69.3%		89.2%		63.1%	
Primary claw II	24.5	*45.4*	15.4	*25.8*	25.4	*55.5*
Secondary claw II	16.0	*29.6*	13.3	*22.2*	15.2	*32.6*
Secondary: primary claw II ratio	65.3%		86.4%		59.8%	
Primary claw III	26.3	*48.7*	16.5	*27.6*	27.2	*57.7*
Secondary claw III	16.4	*30.4*	14.2	*23.8*	16.4	*35.2*
Secondary: primary claw III ratio	62.4%		86.1%		60.3%	
Primary claw IV	33.6	*62.2*	18.9	*31.6*	36.5	*78.3*
Secondary claw IV	20.5	*38.0*	15.4	*25.8*	21.5	*46.2*
Secondary: primary claw IV ratio	61.0%		81.5%		58.9%	

In addition to the body size, the new species differs from *Milnesium eurystomum* and *Milnesium bohleberi* by having a cylindrical (not funnel-shaped) buccal tube; from *Milnesium eurystomum* by having a higher *pt* of the insertion point of the stylet supports (*pt* = 63–66 in *Milnesium minutum* sp. n. vs 58–61 in *Milnesium eurystomum*); and from *Milnesium bohleberi* in having lower percent ratio between the secondary claw and the primary claw lengths on all legs (the percent ratio is 69.7–72.6 in the claws I–III of *Milnesium minutum* sp. n. and 77.9–84.9, for *Milnesium bohleberi* (according to Bartels et al. 2014); in claw IV the values are 65.6–68.9 in *Milnesium minutum* sp. n. and, 78.9–80.4 for *Milnesium bohleberi* (see: Bartels et al. 2014)).

*Milnesium minutum* sp. n. differs from *Milnesium brachyungue* by having slightly lower *pt* of the stylet supports insertion point (63–66 in the new species vs 67–70 in *Milnesium brachyungue*), by higher *pt* of the primary and the secondary claw lengths, and by higher values of the percent ratio between the secondary claw and primary claw lengths (Tables 1 and 2, Figs 1 and 2B).

The new species differs from *Milnesium longiungue* by having accessory points as well as lower *pt* of the primary claw lengths and higher values of the percent ratio between the secondary claw and primary claw lengths (Tables 1 and 2, Figs 1 and 2C).

The new species differs from *Milnesium antarcticum* by having a higher *pt* of the buccal tube width (38.6–42.4 in *Milnesium minutum* sp. n., 25.9-31.8 in *Milnesium antarcticum* according to Tumanov 2006); lower *pt* of the insertion point of the stylet supports on the buccal tube (63.0–66.0 in the new species, 70.0-73.7 in *Milnesium antarcticum* according to Tumanov 2006); higher *pt* of the primary claw lengths on legs I-III (Tables 1 and 4, Figs 1 and 2D).

*Milnesium minutum* sp. n. differs from *Milnesium zsalakoae* by the more anterior insertion of the stylet supports on the buccal tube (*pt* = 63–66 in *Milnesium minutum* sp. n., 68.2–71.1 in *Milnesium zsalakoae*, according to Meyer and Hinton 2010). The new species also differs by having accessory points and by having a higher percent ratio between the secondary claw and primary claw lengths on legs IV where the values are 65.6–68.9 in *Milnesium minutum* and 47.2–48.6 for *Milnesium zsalakoae* (see: Meyer and Hinton 2010).

The new species clearly differs from *Milnesium barbadosense* by having eyes and by having the stylet supports inserted on the buccal tube in a more anterior position (*pt* = 63–66 in the new species, about 73 for *Milnesium barbadosense* according to Meyer and Hinton 2012) (Tables 1 and 4).

### Milnesium sandrae sp. n.

Taxon classificationAnimaliaApochelaMilnesiidae

http://zoobank.org/D17FD526-0722-4D6E-A50A-F855F68110A6

Fig. 3 , Table 3

#### Locus typicus.

Hawaiian Archipelago: Hawai’i Island.

#### Material examined.

Hawaiian Archipelago: Hawai’i Island (holotype, slide 4290) and 16 paratypes (slides Nos. 4268, 4288–4290; 4293) collected in 1994 by Dr. D.S. Horning (Sydney).

The precise geographic coordinates relative to the type locality in which the specimens were found in 1994 are not available. The specimens were erroneously considered as *Milnesium tardigradum* by Binda and Pilato (1994).

#### Type repository.

Holotype and paratypes are deposited in the Binda and Pilato Collection, Museum of the Department of Biological, Geological and Environmental Sciences, University of Catania, Sicily.

#### Specific diagnosis.

Colourless; cuticle smooth; eye spots present; six peribuccal and two lateral papillae present; bucco-pharyngeal apparatus of the *Milnesium* type. Buccal tube wide; mouth terminal with six peribuccal lamellae. Stylet supports inserted on the buccal tube at 58.0–60.5 % of its length. Claws of the *Milnesium* type with [3-3]-[3-3] configuration; primary claws with thin accessory points; secondary claws each with a rounded basal thickening (lunule); a long cuticular bar present under the claws I–III.

#### Description of the holotype.

**Figure 3.**
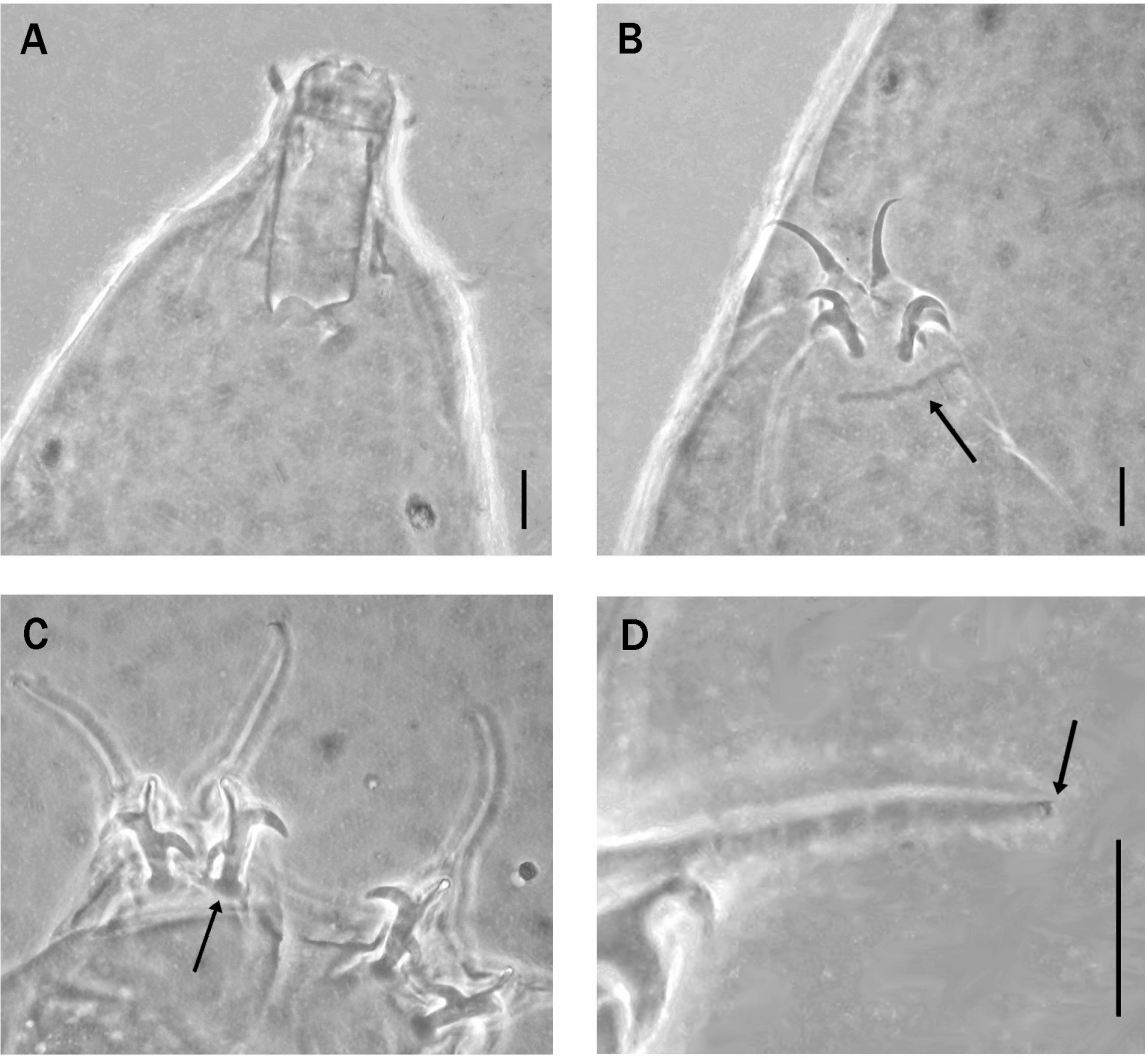
**A–D**, *Milnesium sandrae* sp. n. **A** Bucco-pharyngeal apparatus (holotype) **B** Claws of the first pair of legs; the arrow indicates the long cuticular thickening (holotype) **C** Claws of the hind legs; the arrow indicates a claw basal thickening (lunule) (slide No. 1028) **D** Detail of one claw of the hind legs with an arrow that indicates one accessory point (holotype). Scale bars: 10 µm.

Body 567 µm long, colourless, cuticle smooth without pseudopores, reticulum, tubercles or gibbosities; eye spots present. Six peribuccal and two lateral papillae present. Bucco-pharyngeal apparatus of the *Milnesium* type (Fig. 3A) (rigid buccal tube without ventral lamina, apophyses for the insertion of the stylet muscles in the shape of very short and flat ridges symmetrical with respect to the frontal plane and without caudal processes; pharyngeal bulb elongated, pear-shaped, without apophyses, placoids or septulum); mouth terminal with six triangular peribuccal lamellae with basal stripes. Stylet furcae triangular in shape (Fig. 3A). Buccal tube cylindrical, 35.0 µm long; the external width at the level of the stylet supports insertion point is 15.7 µm (*pt* = 44.9). Stylet supports inserted on the buccal tube at 58.0% of its length.

Claws of the *Milnesium* type (Fig. 3B–D), secondary claws with three points: configuration [3-3]-[3-3]. Primary claws on legs I, 14.5 µm long (*pt* = 41.4), and secondary claw, 12.4 µm (*pt* = 35.4); primary claws on legs II, 15.2 µm long (*pt* = 43.4) and secondary claw, 12.4 µm (*pt* = 35.4); primary claws on legs III, 15.2 µm long (*pt* = 43.4) and secondary claw, 12.2 µm (*pt* =34.9); primary claws on legs IV, 19.2 µm long (*pt* = 54.9) and secondary claw, 13.7 µm (*pt* = 39.1). The secondary claw length is 85.5% of the primary claw length on legs I, 81.6% on legs II, 80.3% on legs III and 71.4% on legs IV.

Thin accessory points present on the primary claws (Fig. 3C, D); secondary claws each with rounded basal thickening (lunule) (Fig. 3C); a long cuticular bar is present under the claws I–III (Fig. 3B).

#### Remarks.

The paratypes are similar to the holotype in both qualitative and quantitative characters (Table 3).

**Table 3.** Measurements in µm, *pt* index values relative to some structures, and percent ratio between secondary claw and primary claw lengths of the holotype, and three paratypes of *Milnesium sandrae* sp. n. Also the differences between maximum and minimum values of some characters are given.

Slide nomber Measurements	4290 Hawai’i Island paratype		4290 Hawai’i Island paratype		4293 Hawai’i Island paratype			4290 Hawai’i Island holotype		Difference between Max.-Min. values
	µm	*pt*	µm	*pt*	µm		*pt*	µm	*pt*	
Body length	401	-	504	-	522		-	567	-	166
Buccal tube length	28.6	-	33.3	-	36.3		-	35.0	-	
Buccal tube width	13.7	*47.9*	16	*48.0*	16.4		*45.2*	15.7	*44.9*	
Stylet supports insertion point		*60.5*		*58.6*			*58.5*		*58.0*	2.5
Primary claw I	?	?	14.5	*43.5*	14.1		*38.8*	14.5	*41.4*	
Secondary claw I	9.7	*33.9*	?	?	11.9		*32.8*	12.4	*35.4*	
Secondary: primary claw I ratio	?		?		84.4%			85.5%		1.1
Primary claw II	12.5	*43.7*	15.5	*46.6*	15.4		*42.4*	15.2	*43.4*	
Secondary claw II	10.3	*36.0*	12.6	*37.8*	12.1		*33.3*	12.4	*35.4*	
Secondary: primary claw II ratio	82.4%		81.3%		78.6%			81.6%		3.8
Primary claw III	13.2	*46.1*	15.0	*45.0*	15.8		*43.5*	15.2	*43.4*	
Secondary claw III	10.5	*36.7*	12.2	*36.6*	12.6		*34.7*	12.2	*34.9*	
Secondary: primary claw III ratio	79.5%		81.3%		79.7%			80.3%		1.8
Primary claw IV	?	?	19.0	*57.1*	19.6	*54.0*		19.2	*54.9*	
Secondary claw IV	?	?	13.4	*40.2*	13.8	*38.0*		13.7	*39.1*	
Secondary: primary claw IV ratio	?		70.5%		70.4%			71.4%		1.0

#### Etymology.

The specific name *sandrae* is in honour of Dr. Sandra J. McInnes (Cambridge, United Kingdom), who kindly improved the English of many of our papers.

#### Differential diagnosis.

*Milnesium sandrae* sp. n. is compared with other species of the genus having six peribuccal lamellae, smooth cuticle (without pseudopores, reticulum, tubercles or gibbosities), and the [3-3]-[3-3] claw configuration. The new species differs from all these species, except *Milnesium eurystomum*, by having a different value of the *pt* index of the stylet supports insertion point (58.0–60.5 in the new species, over 62 in the remaining taxa) and other characters, which are indicated in detail in the following comparisons.

*Milnesium sandrae* sp. n. differs from *Milnesium eurystomum* and *Milnesium bohleberi* by having a cylindrical instead of a funnel-shaped buccal tube.

*Milnesium sandrae* sp. n. differs from *Milnesium brachyungue* by a higher buccal tube width *pt* index; a higher *pt* of both the primary and secondary claw lengths, and lower percent ratio values between the secondary claw and primary claw lengths (Tables 3 and 2, and Figs 3BC and 2B); this ratio difference is particularly marked for legs IV where the ratio values of 70.4–71.4 for *Milnesium sandrae* sp. n. compare with 81 in *Milnesium brachyungue* (Tables 2 and 3).

The new species differs from *Milnesium asiaticum* by having a higher *pt* of the buccal tube width; a higher *pt* of the secondary claw lengths (particularly on the legs I–III), and a higher percent ratio between the secondary claw and primary claw lengths on all legs (Tables 2 and 3).

*Milnesium sandrae* sp. n. differs from *Milnesium antarcticum* by having a shorter buccal tube with respect to the body length; a higher *pt* index of the buccal tube width; higher *pt* of the insertion point of the stylet supports on the buccal tube (58.0-60.5 in *Milnesium sandrae* sp. n., 70.0–73.7 in *Milnesium antarcticum* according to Tumanov 2006); higher *pt* indices of the secondary claws, and higher values of the percent ratio between the secondary claw and primary claw lengths (Tables 3 and 4, Figs 3B, C and 2D).

**Table 4.** Measurements in µm, *pt* index values relative to some structures, and percent ratio between secondary claw and primary claw lengths of the holotype of *Milnesium barbadosense* (*According to Meyer and Hinton 2012) and the holotype of *Milnesium antarcticum* (** according to Tumanov 2006).

Species Measurements	*Milnesium barbadosense*		*Milnesium antarcticum*	
	holotype *		holotype **	
	µm	*pt*	µm	*pt*
Body length	686.4	-	?	-
Buccal tube length	44.0	-	74.7	-
Buccal tube width	21.7	*49.3*	27.4	*36.7*
Stylet supports insertion point		*72.8*		*71.3*
Primary claw I	17.8	*40.5*	26.3	*35.2*
Secondary claw I	12.3	*28.0*	17.8	*23.8*
Secondary: primary claw I ratio	69.1%		67.7%	
Primary claw II	21.6	*49.1*	?	?
Secondary claw II	14	*31.8*	?	?
Secondary: primary claw II ratio	64.8%		?	
Primary claw III	21.1	*48.0*	?	?
Secondary claw III	12.3	*28.0*	?	?
Secondary: primary claw III ratio	58.3%		?	
Primary claw IV	23.3	*53.0*	39.2	*52.5*
Secondary claw IV	16.0	*36.4*	23.7	*31.7*
Secondary: primary claw IV ratio	68.7%		60.5	

The new species differs from *Milnesium longiungue* by having accessory points; by having lower *pt* values of the primary claw, and by a higher percent ratio between the secondary claw and primary claw lengths on all legs (Tables 3 and 4).

The new species differs from *Milnesium zsalakoae* in having accessory points and a higher percent ratio between the secondary claw and primary claw lengths on all legs. The difference is particularly marked in claws IV where the *pt* ratios are 70.4–71.4 in *Milnesium sandrae* sp. n. and 47.2–48.6 in *Milnesium zsalakoae* (see: Meyer and Hinton 2010).

*Milnesium sandrae* sp. n. differs from *Milnesium barbadosense* by higher *pt* of the secondary claw lengths and by higher values of the percent ratio between the secondary claw and the primary claw lengths on legs I–III (Tables 3 and 4).

*Milnesium sandrae* sp. n. differs from *Milnesium minutum* by having a larger body size; shorter buccal tube with respect to the body length; a higher *pt* of the secondary claw lengths and higher values of the percent ratio between the secondary claw and primary claw lengths. This difference is less marked in legs IV (Tables 1 and 3; Figs 1 and 3).

## Conclusions

The description of two new species, *Milnesium minutum* sp. n. and *Milnesium sandrae* sp. n., raises the number of species ascribed to the genus *Milnesium* to 31 (30 living and one fossil). Therefore, this tardigrade genus, considered monospecific for 150 years (1840–1990), today is among the 10 most species rich genera. The first species described, *Milnesium tardigradum* Doyère, 1840, was considered cosmopolitan, but it is evident that specimens of many species have been erroneously attributed to *Milnesium tardigradum* and, therefore, its geographic distribution must be re-examined and it is probable that the distribution of *Milnesium tardigradum* is much smaller than formerly believed (Michalczyk et al. 2012a). Many of the newly described species of *Milnesium* have been reported from only one locality, but it is possible that some of them will be recognized in the future in other geographic areas. Therefore the actual geographic distribution of many species of *Milnesium* has to be considered provisional.

## Supplementary Material

XML Treatment for Milnesium minutum

XML Treatment for Milnesium sandrae

## References

[B1] Bartels PJ Nelson DR Kaczmarek Ł Michalczyk Ł (2014) The genus *Milnesium* (Tardigrada: Eutardigrada: Milnesiidae) in the Great Smoky Mountains National Park (North Carolina and Tennessee, USA) with the description of *Milnesium bohleberi* sp. n. Zootaxa 3826(2): 356–368. doi: 10.11646/zootaxa.3826.2.5 24990052 10.11646/zootaxa.3826.2.5

[B2] Binda G Pilato G (1990) Tardigradi della Terra del Fuoco e Magallanes. *Milnesium brachyungue*, nuova specie di tardigrado Milnesiidae. Animalia 17: 105–110.

[B3] Binda MG Pilato G (1994) Notizie sui Tardigradi delle Isole Hawaii con descrizione di due specie nuove. Animalia 21: 57–62.

[B4] Doyère LMF (1840) Mémoire sur les Tardigrades. I.Annales des Sciences Naturelles (Paris S. 2) 14: 269–362.

[B5] Maucci W (1991) Tre nuove specie di Eutardigradi della Groenlandia Meridionale. Bollettino del Museo Civico di Storia Naturale di Verona 15: 279–289.

[B6] Meyer HA Hinton JG (2010) *Milnesium zsalakoae* and *Milnesium jacobi*, two new species of Tardigrada (Eutardigrada: Milnesiidae) from the southwestern USA. Proceedings of the Biological Society of Washington 123(2): 113–120. doi: 10.2988/09-29.1

[B7] Meyer HA Hinton JC (2012) Terrestrial Tardigrada of the island of Barbados in the West Indies, with the description of *Milnesium barbadosense* sp. n. (Eutardigrada: Apochela: Milnesiidae). Caribbean Journal of Science 46(2–3): 194–202.

[B8] Michalczyk Ł Wełnicz W Frohme M Kaczmarek Ł (2012) Redescription of *Milnesium* Doyère, 1840 taxa (Tardigrada: Eutardigrada: Milnesiidae), including the nominal species for the genus. Zootaxa 3154: 1–20.

[B9] Michalczyk Ł Wełnicz W Frohme M Kaczmarek Ł (2012b) Corrigenda of Zootaxa, 3154: 1–20 Redescriptions of three *Milnesium* Doyère, 1840 taxa (Tardigrada: Eutardigrada: Milnesiidae), including the nominal species for the genus. Zootaxa 3154: 1–20.

[B10] Pilato G (1981) Analisi di nuovi caratteri nello studio degli Eutardigradi. Animalia 8: 51–57.

[B11] Tumanov DV (2006) Five new species of the genus *Milnesium* (Tardigrada, Eutardigrada, Milnesiidae). Zootaxa 1122: 1–23.

